# Mobile colistin resistance *mcr-4.3*- and *mcr-4.6*-harbouring plasmids in livestock- and human-retrieved Enterobacterales in the Netherlands

**DOI:** 10.1093/jacamr/dlad053

**Published:** 2023-05-03

**Authors:** Casper Jamin, Michael S M Brouwer, Kees T Veldman, Erik Beuken, Sandra Witteveen, Fabian Landman, Edou Heddema, Paul H M Savelkoul, Lieke van Alphen, Antoni P A Hendrickx, The Dutch CPE Surveillance Study Group, A Maijer-Reuwer, M A Leversteijn-van Hall, W van den Bijllaardt, R van Mansfeld, K van Dijk, B Zwart, B M W Diederen, J W Dorigo-Zetsma, D W Notermans, A Ott, W Ang, J da Silva, A L M Vlek, A G M Buiting, L G M Bode, S Paltansing, A J van Griethuysen, M J C A van Trijp, M den Reijer, M Wong, A E Muller, M P M van der Linden, M van Rijn, S B Debast, K Waar, E Kolwijck, N Al Naiemi, T Schulin, S Dinant, S P van Mens, D C Melles, M P A van Meer, J W T Cohen Stuart, P Gruteke, I T M A Overdevest, A van Dam, I Maat, B Maraha, J C Sinnige, E E Mattsson, N van Maarseveen, E de Jong, S J Vainio, E Heikens, R Steingrover, A Troelstra, E Bathoorn, J de Vries, D W van Dam, E I G B de Brauwer, T Halaby, H Berkhout

**Affiliations:** Department of Medical Microbiology, Infectious Diseases & Infection Prevention, Care and Public Health Research Institute (CAPHRI), Maastricht University Medical Center, Maastricht, The Netherlands; Wageningen Bioveterinary Research, Lelystad, The Netherlands; Wageningen Bioveterinary Research, Lelystad, The Netherlands; Department of Medical Microbiology, Infectious Diseases & Infection Prevention, Care and Public Health Research Institute (CAPHRI), Maastricht University Medical Center, Maastricht, The Netherlands; Center for Infectious Disease Control (CIb), National Institute for Public Health and the Environment (RIVM), Bilthoven, The Netherlands; Center for Infectious Disease Control (CIb), National Institute for Public Health and the Environment (RIVM), Bilthoven, The Netherlands; Department of Medical Microbiology and Infection Control, Zuyderland Medical Center, Sittard-Geleen/Heerlen, The Netherlands; Department of Medical Microbiology, Infectious Diseases & Infection Prevention, Care and Public Health Research Institute (CAPHRI), Maastricht University Medical Center, Maastricht, The Netherlands; Department of Medical Microbiology and Infection Control, Amsterdam UMC, Location VUmc, Amsterdam, The Netherlands; Department of Medical Microbiology, Infectious Diseases & Infection Prevention, Care and Public Health Research Institute (CAPHRI), Maastricht University Medical Center, Maastricht, The Netherlands; Center for Infectious Disease Control (CIb), National Institute for Public Health and the Environment (RIVM), Bilthoven, The Netherlands

After the first report of the mobile colistin resistance gene *mcr-1* in 2015, 10 *mcr* variants were identified among antibiotic-resistant Enterobacterales and were mostly located on plasmids.^1,2^ The *mcr* genes encode phosphoethanolamine transferases capable of modifying Lipid A in membrane-associated LPS, thereby ultimately leading to colistin resistance.^3^ Colistin is considered a last-resort antibiotic for treatment of human Enterobacterales infections, but was also being used in veterinary medicine in the Netherlands in 2021.^4^ Until now, the presence of *mcr-4* has not been described before in The Netherlands. The major objective of this study was to analyse *mcr-4*-encoding plasmids from Enterobacterales obtained from humans and livestock in the Netherlands. To address this, a search for *mcr-4-*containing Enterobacterales isolates was performed in two surveillance databases containing Illumina short-read next-generation sequencing (NGS) data. These include the carbapenemase-producing Enterobacterales (CPE) surveillance collection from the National Institute for Public Health and the Environment (RIVM) and the livestock resistance monitoring collection from Wageningen Bioveterinary Research (WBVR). In addition, two clinical isolates of the Zuyderland Medical Center (ZMC) were analysed by the Maastricht University Medical Centre (MUMC).

The national CPE surveillance collection of the RIVM was searched for unique CPE and non-CPE isolates in the period from 2012 until 2020, in which only the first bacterial species and carbapenemase allele combination per person were included, yielding 3008 Enterobacterales isolates. This revealed two *Enterobacter* spp. (0.07%) with MLST ST54 that carried *mcr-4.3*. Average nucleotide identity (ANI) calculation using http://enve-omics.ce.gatech.edu/ani/index of the *Enterobacter* sp. ST54 with *Enterobacter kobei* strain UCI 23 (GenBank accession NZ_KI973153.1) revealed an ANI of 99.09%, demonstrating that the *mcr-4.3*-carrying isolates were *E. kobei*. The collection of the WBVR in the period from 2018 until 2021 revealed that 4 out of 1200 veal calf isolates (0.3%) harboured *mcr-4.6*. Three *mcr-4.6*-containing plasmids, one from *Hafnia paralvei* and two from *Escherichia coli* were included in this study. The collection of the ZMC contained two *E. kobei* ST54 isolates with *mcr-4.3* isolated from hospitalized patients. Isolates were also subjected to Nanopore long-read sequencing by different in-house methods.^5,6^ Hybrid assembly of short-read and long-read sequencing data was performed using Unicycler (v.0.4.8, default settings) to reconstruct *mcr-4* plasmids, which were compared to internationally reported *mcr-4* plasmid sequences from NCBI (accessed 14 October 2022) using BioNumerics (v7.6.3).^7^ Hybrid *mcr-4* plasmid assemblies are available in GenBank (BioProject accession number PRJNA941496). Colistin resistance was confirmed using a standardised broth microdilution (BMD; Micronaut MIC strip colistin, Merlin), as previously described.^8^

Seven isolates carried *mcr-4*-like alleles and had varying resistomes (Figure 1a). In addition to *mcr-4.3*, the RIVM_C009363 isolate contained the *ant(2′′)-Ia*, *bla*_ACT-9_-like, *bla*_CTX-M-9_, *fosA*, *mcr-9.1*, *qnrA1*, *sul1* and *tet*(A) resistance genes and lacked a carbapenemase gene. The RIVM_C014549 isolate was multi-resistant, containing the *aac(6′)-Ib-cr*, *aac(6′)-Ib3*, *aadA1*, *aph(3′′)-Ib*, *aph(3′)-XV*, *aph(6)-Id*, *bla*_ACT-9_, *bla*_SHV-12_, *bla*_VIM-1_, *catB2*, *dfrA14*, *fosA*, *mcr-9.1*, *mph*(A), *qnrS1* and *sul1/2* genes. The *E. kobei* isolates MUMC-1 and MUMC-2 also belonged to MLST ST54, but were not clonally related to the RIVM_C014549 and RIVM_C009363 *E. kobei* isolates since they differed by 191 to 270 SNPs as inferred using split K-mer analysis using default settings. These isolates contained the *mcr-4.3*, aph(6)-Id*, aph(3′′)-Ib*, sul2*, catA1*, *bla*_CTX-M-15_*, *dfrA14*, *oqxA10*, *oqxB4*, *bla*_OXA-1_, *aac(6′)-Ib-cr*, *aac(3)-IIe*, aadA1*, *mcr-9.1*, *qnrB1*, *bla*_ACT-64_, *tet*(A) (*absent in MUMC-1) resistance genes. The livestock *E. coli* isolates (WUR-NRS20181383 and WUR-NRS20181408) were from ST216, and contained the *aadA1*, *bla*_TEM-1_, *bla*_EC-15_, *dfrA1*, *sul1* and *tet*(B) resistance genes. The *H. paralvei* isolate (WUR-NRS20180341) contained the *aadA1*, *bla*_ACC-1b_, *mph*(B), *sul1*, *dfrA1*, *mcr-9.1* and *tet*(B) genes. None of the seven isolates carrying *mcr-4* contained mutations in the *pmrAB* genes, known to be associated with colistin resistance, as inferred using PointFinder v4.1.^9^

**Figure 1.**
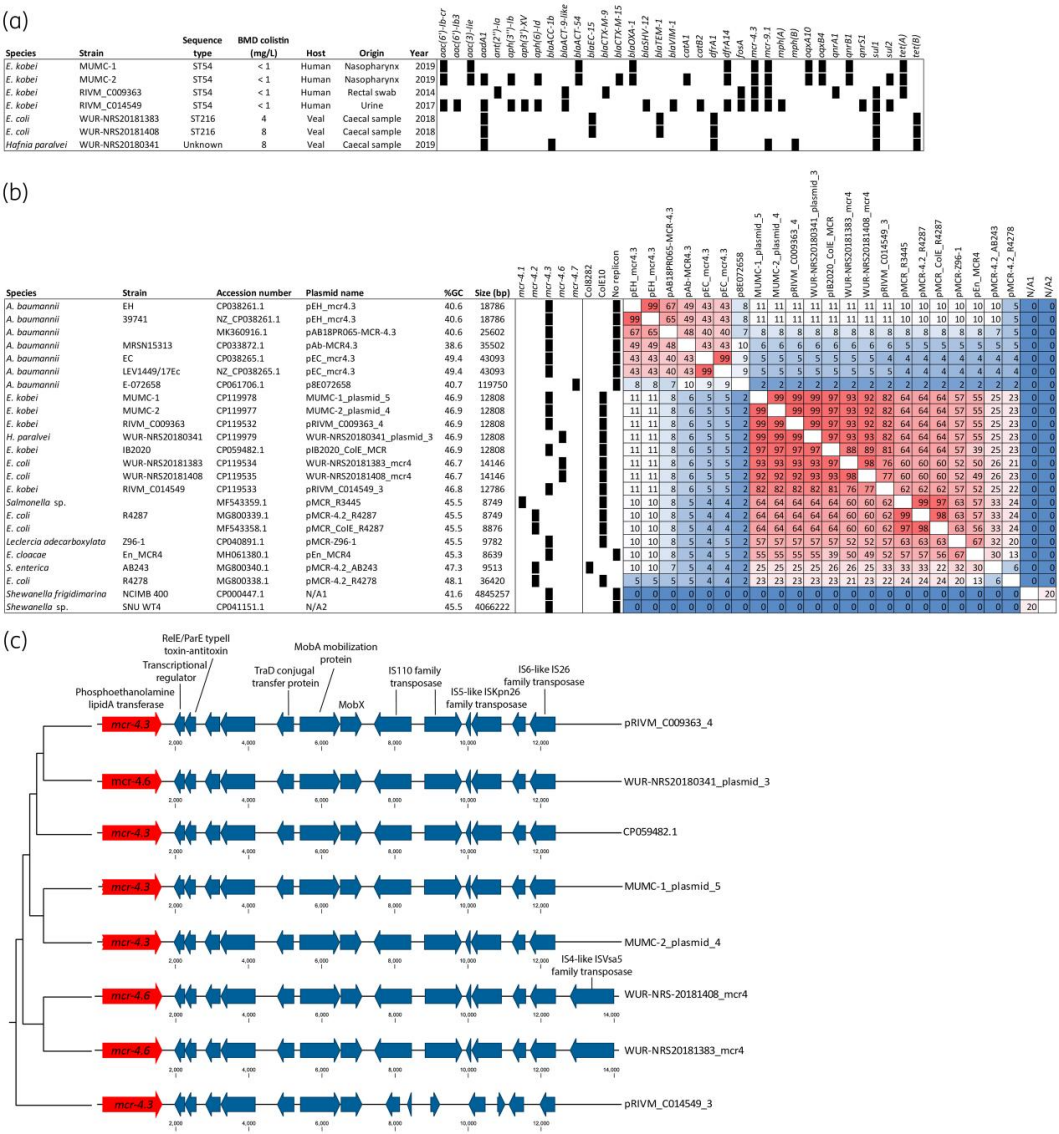
*In silico* characterization of *mcr-4*-containing plasmids. (a) Short-read sequencing-based molecular characteristics of the *mcr-4*-carrying isolates analysed in this study. (b) Comparison of *mcr-4* plasmids from the Netherlands in context with NCBI-retrieved plasmids. The % G + C content and plasmid size in bp is indicated. The presence of *mcr-4* alleles and replicons is indicated by squares. Heatmap indicating percentage identity. (c) Alignment of all *mcr-4*-harbouring plasmids from the Netherlands with one *E. kobei* plasmid (pIB2020_ColE_MCR, CP059482.1) from Italy using CLC Genomics Workbench (v22.0.2). Plasmids were rotated and the *mcr-4* allele was set as starting point for comparison.

Seven highly similar (76%–99%) and small (12.8–14.1 kb) *mcr-4* plasmids with a ColE10 replicon were identified with either an *mcr-4.3* allele in four human *E. kobei* isolates (MIC colistin ≤1 mg/L), or an *mcr-4.6* allele in three livestock-associated isolates (MIC colistin 4–8 mg/L) collected between 2012 and 2021 (Figure 1b). More specifically, the MUMC-1_plasmid_5, MUMC-2_plasmid_4 and pRIVM_C009363_4 were 99% identical plasmids of 12.8 kb with a %G + C of 46.92%. The WUR-NRS20180341_plasmid_3, WUR-NRS20181383_mcr4 and WUR-NRS-20181408_mcr4 had slightly different %G + C and an *mcr-4.6* allele possibly conferring resistance to colistin. Four human *E. kobei* isolates (MUMC-01, MUMC-02, RIVM_C014549, RIVM_C009363) carried both *mcr-4.3* and *mcr-9*; *mcr-9* is not associated with colistin resistance in *Enterobacter.*^10,11^ Dutch livestock-retrieved isolates with *mcr-4.6* plasmids differed from the human-derived plasmids by one SNP in the *mcr-4.3* gene, leading to an *mcr-4.6* allelic variant, all exhibiting resistance to colistin.^12,13^ The *mcr-4* plasmids identified among Enterobacterales in the Netherlands were distinct from the *mcr-4* plasmids in the NCBI database (Figure 1b). However, one *E. kobei* plasmid (pIB2020_ColE_MCR, CP059482.1) from Italy from 2019 closely resembled the *mcr-4.3* and *mcr-4.6* plasmids from the Netherlands, suggesting international spread.^14^ The Dutch *mcr-4* plasmid architecture was comparable and comprised the phosphoethanolamine lipid A transferase gene *mcr-4*, followed by a type II toxin/antitoxin system, TraD conjugal transfer protein, MobA/X mobilization proteins and transposases (Figure 1c). The pRIVM_C014549_3 was different from the other *mcr-4* plasmids obtained in the Netherlands.

To test *mcr-4-*mediated colistin resistance, two strategies were employed.

Firstly, cloning of *mcr-4.3* and *mcr-4.6* with and without native promotor using primers MCR4_ownpromotorF: CACGGGCAAAGATCGGAGGG, MCR4_reverse: TCAGATCTCGTTGTAATTTTCAAGG and MCR4_withoutownpromotorF: GAGGTCAAGCTTGTATTGTTTTT into pGEM^®^-T Easy (Invitrogen) in *E. coli* DH10β was performed according to the manufacturer’s instructions. Plating was done on LB agar plates with 0, 0.125, 0.25, 0.5, 1 or 2 mg/L colistin and with ampicillin (50 mg/L). To avoid overexpression of the *mcr-4* genes, IPTG was omitted in the LB agar plates (no blue–white screening). No colonies were found after overnight incubation at 37°C on plates containing colistin. Also, induction with IPTG for the native and non-native *mcr-4* promotor constructs, did not result in colistin resistant colonies. Seven of the eight selected colonies that grew on LB agar plates with only ampicillin showed inverted *mcr-4* inserts, as determined by sequencing.

Secondly, transformation of an *Acinetobacter baumannii* (BioSample: ERS7182715, MIC colistin 0.5 mg/L) with *mcr-4.3* and *mcr-4.6* plasmid DNA isolated from isolate MUMC-1 and WUR_NRS20181383, respectively, was performed. After transformation of *A. baumannii* with either *mcr-4.3* or *mcr-4.6* plasmids, only colonies were observed on plates with colistin concentration up to and including 0.25 mg/L, indicating there was no change in colistin resistance for this strain.

In summary, the occurrence of *mcr-4* plasmids among Enterobacterales is low in the Netherlands. The *mcr-4.3* allele in *E. kobei* and the *mcr-4.6* allele in livestock *E. coli* and *H. paralvei* likely do not encode for colistin resistance in the human and livestock isolates. A recent study also failed to detect colistin resistance in *E. kobei* ST54 co-harbouring *mcr-4.3* and *mcr-9* and is in line with this study.^15^ In contrast, *mcr-4.3* conferred colistin resistance in *A. baumannii* and *Acinetobacter nosocomialis*,^16,17^ suggesting species-specific functionality of this colistin resistance gene, but could not be confirmed in the *A. baumannii* isolate analysed in this study. The *mcr-4* plasmids from human and livestock were virtually identical, suggesting unnoticed horizontal dissemination of these plasmids in the Netherlands.

## Members of the Dutch CPE Surveillance Study Group

A. Maijer-Reuwer, ADRZ medisch centrum, Department of Medical Microbiology, Goes; M. A. Leversteijn-van Hall, Alrijne Hospital, Department of Medical Microbiology, Leiden; W. van den Bijllaardt, Amphia Hospital, Microvida Laboratory for Microbiology, Breda; R. van Mansfeld, Amsterdam UMC—location AMC, Department of Medical Microbiology and Infection Control, Amsterdam; K. van Dijk, Amsterdam UMC—location VUmc, Department of Medical Microbiology and Infection Control, Amsterdam; B. Zwart, Atalmedial, Department of Medical Microbiology, Amsterdam; B. M. W. Diederen, Bravis Hospital/ZorgSaam Hospital Zeeuws-Vlaanderen, Department of Medical Microbiology, Roosendaal/Terneuzen; J. W. Dorigo-Zetsma, TergooiMC, Central Bacteriology and Serology Laboratory, Hilversum; D. W. Notermans, Centre for Infectious Disease Control, National Institute for Public Health and the Environment, Bilthoven; A. Ott, Certe, Medical Microbiology Groningen, Drenthe, Groningen; W. Ang, Comicro, Department of Medical Microbiology, Hoorn; J. da Silva, Deventer Hospital, Department of Medical Microbiology, Deventer; A. L. M. Vlek, Diakonessenhuis Utrecht, Department of Medical Microbiology and Immunology, Utrecht; A. G. M. Buiting, Elisabeth-TweeSteden (ETZ) Hospital, Department of Medical Microbiology and Immunology, Tilburg; L. G. M. Bode, Erasmus University Medical Center, Department of Medical Microbiology and Infectious Diseases, Rotterdam; S. Paltansing, Franciscus Gasthuis & Vlietland, Department of Medical Microbiology and Infection Control, Rotterdam; A. J. van Griethuysen, Gelderse Vallei Hospital, Department of Medical Microbiology, Ede; M. den Reijer, Star-shl diagnostic centre, Department of Medical Microbiology, Rotterdam; M. J. C. A. van Trijp, Groene Hart Hospital, Department of Medical Microbiology and Infection Prevention, Gouda; M. Wong, Haga Hospital, Department of Medical Microbiology, ‘s-Gravenhage; A. E. Muller, HMC Westeinde Hospital, Department of Medical Microbiology, ‘s-Gravenhage; M. P. M. van der Linden, IJsselland hospital, Department of Medical Microbiology, Capelle a/d Ijssel; M. van Rijn, Ikazia Hospital, Department of Medical Microbiology, Rotterdam; S. B. Debast, Isala Hospital, Laboratory of Medical Microbiology and Infectious Diseases, Zwolle; K. Waar, Certe, Medical Microbiology Friesland, Noordoostpolder, Leeuwarden; E. Kolwijck, Jeroen Bosch Hospital, Department of Medical Microbiology and Infection Control, ‘s-Hertogenbosch; N. Al Naiemi, LabMicTA, Regional Laboratory of Microbiology Twente Achterhoek, Hengelo; T. Schulin, Laurentius Hospital, Department of Medical Microbiology, Roermond; S. Dinant, Maasstad Hospital, Department of Medical Microbiology, Rotterdam; S. P. van Mens, Maastricht University Medical Centre, Department of Medical Microbiology, Infectious Diseases & Infection Prevention, Maastricht; D. C. Melles, Meander Medical Center, Department of Medical Microbiology, Amersfoort; M. P. A. van Meer, Rijnstate Hospital, Laboratory for Medical Microbiology and Immunology, Velp; J. W. T. Cohen Stuart, Noordwest Ziekenhuisgroep, Department of Medical Microbiology, Alkmaar; P. Gruteke, OLVG Lab BV, Department of Medical Microbiology, Amsterdam; I. T. M. A. Overdevest, PAMM, Department of Medical Microbiology, Veldhoven; A. van Dam, Public Health Service, Public Health Laboratory, Amsterdam; I. Maat, Radboud University Medical Center, Department of Medical Microbiology, Nijmegen; B. Maraha, Albert Schweitzer Hospital, Department of Medical Microbiology, Dordrecht; J. C. Sinnige, Regional Laboratory of Public Health, Department of Medical Microbiology, Haarlem; E. E. Mattsson, Reinier de Graaf Groep, Department of Medical Microbiology, Delft; N. van Maarseveen, Saltro Diagnostic Centre, Department of Medical Microbiology, Utrecht; E. de Jong, Slingeland Hospital, Department of Medical Microbiology, Doetinchem; S. J. Vainio, St Antonius Hospital, Department of Medical Microbiology and Immunology, Nieuwegein; E. Heikens, St Jansdal Hospital, Department of Medical Microbiology, Harderwijk; R. Steingrover, St. Maarten Laboratory Services, Department of Medical Microbiology, Cay Hill (St. Maarten); A. Troelstra, University Medical Center Utrecht, Department of Medical Microbiology, Utrecht; E. Bathoorn, University of Groningen, Department of Medical Microbiology, Groningen; J. de Vries, VieCuri Medical Center, Department of Medical Microbiology, Venlo; D. W. van Dam, Zuyderland Medical Centre, Department of Medical Microbiology and Infection Control, Sittard-Geleen; E. I. G. B. de Brauwer, Zuyderland Medical Centre, Department of Medical Microbiology and Infection Control, Heerlen; T. Halaby, Analytical Diagnostic Center N.V. Curaçao, Department of Medical Microbiology, Willemstad (Curaçao); H. Berkhout, Canisius Wilhelmina Hospital, Department of Medical Microbiology and Infectious Diseases, Nijmegen.

## References

[1] Ling Z , Yin W, Shen Z et al Epidemiology of mobile colistin resistance genes *mcr-1* to *mcr-9*. J Antimicrob Chemother 2020; 75: 3087–95. 10.1093/jac/dkaa205 32514524

[2] Wang C , Feng Y, Liu L et al Identification of novel mobile colistin resistance gene *mcr-10*. Emerg Microbes Infect 2020; 9: 508–16. 10.1080/22221751.2020.1732231 32116151 PMC7067168

[3] Poirel L , Jayol A, Nordmann P. Polymyxins: antibacterial activity, susceptibility testing, and resistance mechanisms encoded by plasmids or chromosomes. Clin Microbiol Rev 2017; 30: 557–96. 10.1128/CMR.00064-16 28275006 PMC5355641

[4] de Greeff SC , Kolwijck E, Schoffelen AF et al NethMap 2022. Consumption of Antimicrobial Agents and Antimicrobial Resistance Among Medically Important Bacteria in the Netherlands in 2021/MARAN 2022. Monitoring of Antimicrobial Resistance and Antibiotic Usage in Animals in the Netherlands in 2021. 2022. http://hdl.handle.net/10029/625885.

[5] Hendrickx APA , Landman F, de Haan A et al *bla* _OXA-48_-like genome architecture among carbapenemase-producing *Escherichia coli* and *Klebsiella pneumoniae* in the Netherlands. Microb genomics 2021; 7: 000512. 10.1099/mgen.0.000512 PMC8209719 33961543

[6] Jamin C , Sanders BK, Zhou M et al Genetic analysis of plasmid-encoded *mcr-1* resistance in Enterobacteriaceae derived from poultry meat in the Netherlands. JAC-Antimicrobial Resist 2021; 3: dlab156. 10.1093/jacamr/dlab156 PMC8597959 34806003

[7] Wick RR , Judd LM, Gorrie CL et al Unicycler: resolving bacterial genome assemblies from short and long sequencing reads. PLoS Comput Biol 2017; 13: e1005595. 10.1371/journal.pcbi.1005595 PMC5481147 28594827

[8] Vendrik KEW , de Haan A, Witteveen S et al A prospective matched case-control study on the genomic epidemiology of colistin-resistant Enterobacterales from Dutch patients. Commun Med 2022; 2: 55. 10.1038/s43856-022-00115-6 35607432 PMC9122983

[9] Zankari E , Allesøe R, Joensen KG et al PointFinder: a novel web tool for WGS-based detection of antimicrobial resistance associated with chromosomal point mutations in bacterial pathogens. J Antimicrob Chemother 2017; 72: 2764–8. 10.1093/jac/dkx217 29091202 PMC5890747

[10] Tyson GH , Li C, Hsu CH et al The *mcr-9* gene of *Salmonella* and *Escherichia coli* is not associated with colistin resistance in the United States. Antimicrob Agents Chemother 2020; 64: e00573-20. 10.1128/AAC.00573-20 PMC7526823 32513803

[11] Hendrickx APA , Debast S, Pérez-Vázquez M et al A genetic cluster of MDR *Enterobacter cloacae* complex ST78 harbouring a plasmid containing *bla*_VIM-1_ and *mcr-9* in the Netherlands. JAC Antimicrobial Resist 2021; 3: dlab046. 10.1093/jacamr/dlab046 PMC8210100 34223115

[12] Rebelo AR , Bortolaia V, Kjeldgaard JS et al Multiplex PCR for detection of plasmid-mediated colistin resistance determinants, *mcr-1*, *mcr-2*, *mcr-3*, *mcr-4* and *mcr-5* for surveillance purposes. Euro Surveill 2018; 23: 17-00672. 10.2807/1560-7917.ES.2018.23.6.17-00672 PMC5824125 29439754

[13] Zhang H , Hou M, Xu Y et al Action and mechanism of the colistin resistance enzyme MCR-4. Commun Biol 2019; 2: 36. 10.1038/s42003-018-0278-1 30701201 PMC6347640

[14] Marchetti VM , Bitar I, Sarti M et al Genomic characterization of VIM and MCR co-producers: the first two clinical cases, in Italy. Diagnostics 2021; 11: 79. 10.3390/diagnostics11010079 33418979 PMC7825325

[15] Kim JS , Kwon MJ, Jeon SJ et al Identification of a carbapenem-resistant *Enterobacter kobei* clinical strain co-harbouring *mcr-4.3* and *mcr-9* in Republic of Korea. J Glob Antimicrob Resist 2021; 26: 114–6. 10.1016/j.jgar.2021.05.008 34133988

[16] Martins-Sorenson N , Snesrud E, Xavier DE et al A novel plasmid-encoded *mcr-4.3* gene in a colistin-resistant *Acinetobacter baumannii* clinical strain. J Antimicrob Chemother 2020; 75: 60–4. 10.1093/jac/dkz413 31578567 PMC6910164

[17] Snyman Y , Reuter S, Whitelaw AC et al Characterisation of *mcr-4.3* in a colistin-resistant *Acinetobacter nosocomialis* clinical isolate from Cape Town, South Africa. J Glob Antimicrob Resist 2021; 25: 102–6. 10.1016/j.jgar.2021.03.002 33757821

